# Complete mitochondrial genome of brown-banded butterflyfish *Chaetodon modestus* (Chaetodontiformes, Chaetodontidae) and phylogenetic analysis

**DOI:** 10.1080/23802359.2022.2148490

**Published:** 2022-11-23

**Authors:** Maheshkumar Prakash Patil, Jong-Oh Kim, Yu-Jin Lee, Yong Bae Seo, Jin-Koo Kim, Gun-Do Kim

**Affiliations:** a Industry-University Cooperation Foundation, Pukyong National University, Busan, Republic of Korea; b Department of Microbiology, Pukyong National University, Busan, Republic of Korea; c School of Marine and Fisheries Life Science, Pukyong National University, Busan, Republic of Korea; d Department of Marine Biology, Pukyong National University, Busan, Republic of Korea; e Research Institute for Basic Science, Pukyong National University, Busan, Republic of Korea

**Keywords:** Brown-banded butterflyfish, *Chaetodon modestus*, Chaetodontidae, mitochondrial genome, phylogenetic analysis

## Abstract

The complete mitochondrial genome of the *Chaetodon modestus* (Temminck and Schlegel, 1844) was first determined in this study, which is 16,490 bp in length, containing 13 protein-coding genes, two rRNA genes, and 22 tRNA. Out of 37 mitochondrial genes, except for *ND6* and eight tRNA (*Pro*, *Glu*, *Ser*, *Tyr*, *Cys*, *Asn*, *Ala*, *Gln*) genes were encoded on the L-strand, the others were encoded on the H-strand. The overall base composition includes A (28.0%), T (28.7%), G (16.7%), ad C (26.6%). The phylogenetic tree was built using the maximum-likelihood approach to provide a relationship within Chaetodontidae, which might be valuable for species management.

*Chaetodon modestus* also known as brown-banded butterflyfish is a species of bony fish in the family Chaetodontidae and the order Chaetodontiformes found on coral reefs all over the world. The morphological distinctive characteristics of the *Chaetodon modestus* include a pattern of pale brown vertical bands on the body and an about eye-sized black spot on the dorsal fin (Kuiter 2004). The genus *Chaetodon* is widely distributed and there are many reports suggesting closely related species based on their morphological appearance or based on mitochondrial DNA (mtDNA) cytochrome *b* and 12S rRNA genes (Kuiter 2004; Hsu et al. 2007). However, there is little knowledge of its mitochondrial genetic features. As a result, we focused our research on the analysis of the complete mtDNA of *C. modestus* and its evolutionary connections within Chaetodontidae. The findings of this work might be relevant in future studies on taxonomic resolution, population genetic structure, phylogeography, and phylogenetic relationships.

A specimen of *C. modestus* (Figure 1(a)) was collected from the coast of Namhae, South Korea (34°70′29.63″N 127°88′36.61″E), and deposited at the Marine Fish Resources Bank of Korea (MFRBK) in Pukyong National University (PKNU), Busan, Republic of Korea (Dr. Jin-Koo Kim, taengko@pknu.ac.kr) under the voucher number PKU-61422. From muscle tissue, total DNA was extracted according to the manufacturer’s instructions using the DNeasy Blood and Tissue Kit (Qiagen, Hilden, Germany). The DNA library was made using the TruSeq Nano DNA Kit and sequenced with paired-end reads (150 bp) on the Illumina platform (Illumina HiSeq 2500, San Diego, CA). Next, the SPAdes v3.13.0 assembly tool (Bankevich et al. 2012) was used for *De novo* assembly and the MitoFish web-server (http://mitofish.aori.u-tokyo.ac.jp/) was used for complete mtDNA sequence annotation (Iwasaki et al. 2013). In order to determine the taxonomic status of *C. modestus*, the complete mtDNA sequences of Chaetodontidae members as well as *Salvelinus malma* as an outgroup member were obtained from NCBI (https://www.ncbi.nlm.nih.gov/) and used to build a phylogenetic tree in MEGA11 v11.0.8 using the maximum-likelihood method (Tamura-Nei model with 1000 bootstraps replication) (Tamura et al. 2021).

**Figure 1.**
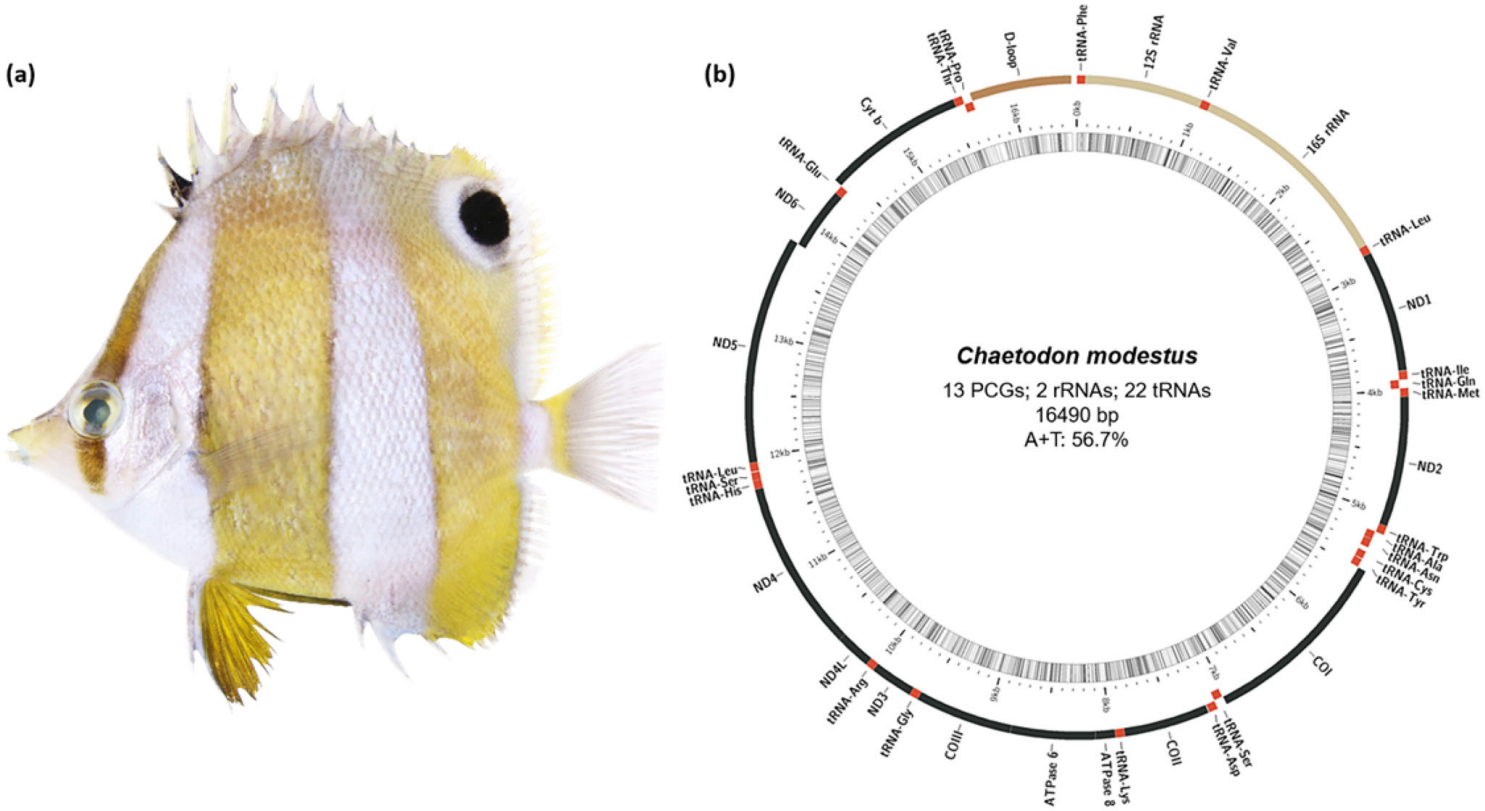
(a) Specimen image of *Chaetodon modestus* (brown-banded butterflyfish) having a characteristic pattern of pale brown vertical bands on the body and about an eye-sized black spot on the dorsal fin. (b) The circular-mapping mitochondrial genome of *C. modestus* was prepared using the MitoFish web server. Genes outside the circle are transcribed clockwise, whereas those inside are transcribed counterclockwise. PCGs: protein-coding genes.

The mtDNA sequence of *C. modestus* has been submitted to GenBank under the accession number ON843631. The close-circular mtDNA of *C. modestus* was 16,490 bp long, containing a total of 37 genes, including 13 protein-coding genes, 22 tRNA genes, two rRNA genes, and one non-coding region (Figure 1(b)). The overall base composition is 28.0%, 28.7%, 16.7%, and 26.6% for A, T, G, and C, respectively, with a slight A + T bias (56.7%) like other vertebrate mitochondrial genomes. Out of 37 mitochondrial genes, except for *ND6* and eight tRNA (*Pro*, *Glu*, *Ser*, *Tyr*, *Cys*, *Asn*, *Ala*, *Gln*) genes were encoded on the L-strand, the others were encoded on the H-strand. The 12S and 16S rRNA genes are situated between the tRNA-Phe and tRNA-Leu genes, separated by the tRNA-Val gene, like in other vertebrates. In the present study, we found that the genes including *ND2*, *COII*, *ND4*, *ND5*, *ND6*, and *Cytb* have an incomplete stop codon. The features mentioned above were in accordance with the typical Chaetodontidae fish mitogenome.

The maximum-likelihood phylogenetic tree based on complete mtDNA sequences of *C. modestus* and other 11 species from Chaetodontidae along with *Salvelinus malma* as an outgroup member was constructed under the Tamura-Nei model with 1000 bootstraps replications (Figure 2). The phylogenetic analysis showed that *C. modestus* is placed in a sister clade to the *C. nippon* with a bootstrap rate of 100%, indicating that they are more closely related. A detailed morphological and molecular phylogenetic analysis is necessary to understand the phylogeny of species. This work describes the complete mitogenome of *C. modestus* and reconstructs the phylogenetic relationship. The complete mtDNA data will be useful in future research on Chaetodontidae species evolution and phylogenetic connections.

**Figure 2.**
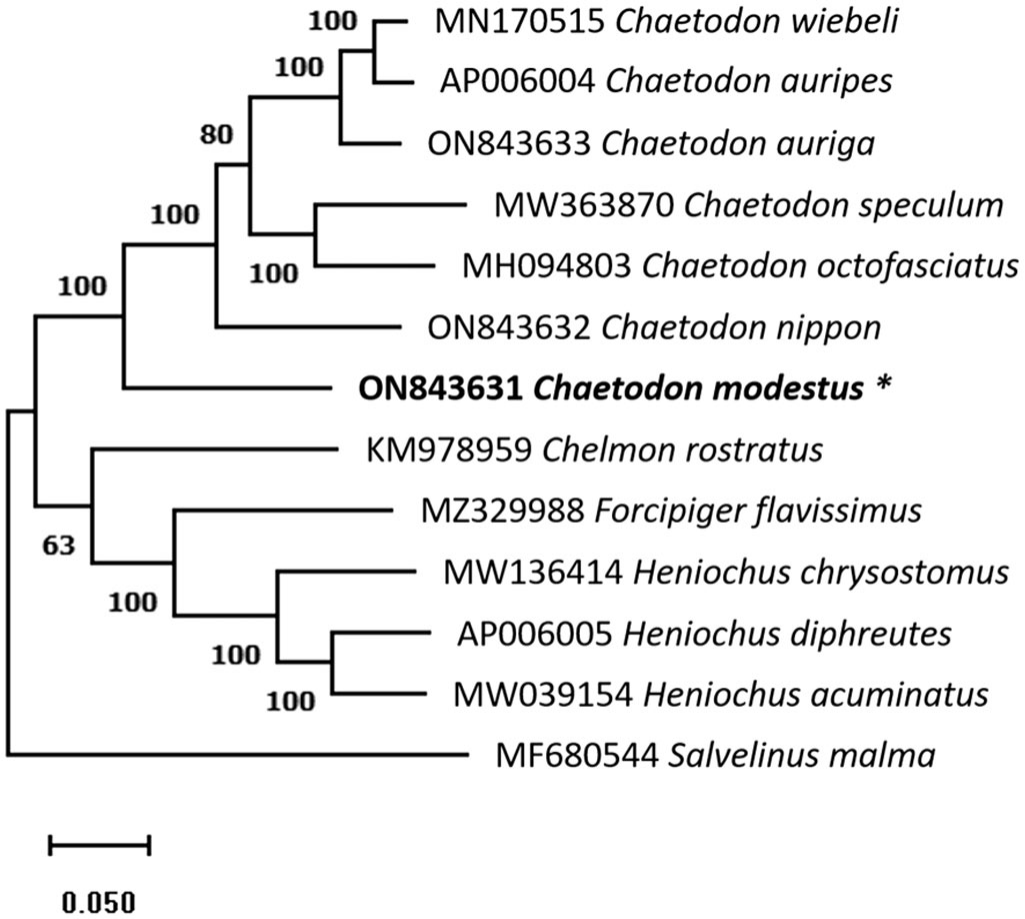
The phylogenetic tree indicates the relationship between *C. modestus* (ON843631) and 11 Chaetodontidae species, based on the mtDNA sequence retrieved from the NCBI database. *Salvelinus malma* as an outgroup member. The mitochondrial genome determined in this work is indicated by an asterisk and the number above the branches indicates the bootstrap value.

## Ethical approval

The sample used for this study was a dead body of fish and as per the animal experimental ethics in the Republic of Korea (Standard Operating Guideline; IACUC – Institutional Animal Care and Use Committee, Book no. 11-1543061-000457-01, effective from December 2020) does not need any approval from Ethics Committee.

## Data Availability

The genome sequence data that support the findings of this study are openly available in GenBank of NCBI at https://www.ncbi.nlm.nih.gov/ under accession no. ON843631. The associated BioProject, BioSample, and SRA numbers are PRJNA854202, SAMN29424364, and SRR19903871, respectively.
